# HIV-1 Group P is unable to antagonize human tetherin by Vpu, Env or Nef

**DOI:** 10.1186/1742-4690-8-103

**Published:** 2011-12-15

**Authors:** Daniel Sauter, Stéphane Hué, Sarah J Petit, Jean-Christophe Plantier, Greg J Towers, Frank Kirchhoff, Ravindra K Gupta

**Affiliations:** 1 Institute of Molecular Virology, Ulm University Medical Center, 89069, Ulm, Germany; 2 Division of Infection and Immunity, University College London, Gower Street, London, WC1E 6BT, UK; 3 Laboratoire associé au Centre National de Référence du Virus de l'Immunodéficience Humaine, Centre Hospitalier Universitaire de Rouen, Rouen, France

## Abstract

**Background:**

A new subgroup of HIV-1, designated Group P, was recently detected in two unrelated patients of Cameroonian origin. HIV-1 Group P phylogenetically clusters with SIVgor suggesting that it is the result of a cross-species transmission from gorillas. Until today, HIV-1 Group P has only been detected in two patients, and its degree of adaptation to the human host is largely unknown. Previous data have shown that pandemic HIV-1 Group M, but not non-pandemic Group O or rare Group N viruses, efficiently antagonize the human orthologue of the restriction factor tetherin (BST-2, HM1.24, CD317) suggesting that primate lentiviruses may have to gain anti-tetherin activity for efficient spread in the human population. Thus far, three SIV/HIV gene products (*vpu, nef *and *env*) are known to have the potential to counteract primate tetherin proteins, often in a species-specific manner. Here, we examined how long Group P may have been circulating in humans and determined its capability to antagonize human tetherin as an indicator of adaptation to humans.

**Results:**

Our data suggest that HIV-1 Group P entered the human population between 1845 and 1989. Vpu, Env and Nef proteins from both Group P viruses failed to counteract human or gorilla tetherin to promote efficient release of HIV-1 virions, although both Group P Nef proteins moderately downmodulated gorilla tetherin from the cell surface. Notably, Vpu, Env and Nef alleles from the two HIV-1 P strains were all able to reduce CD4 cell surface expression.

**Conclusions:**

Our analyses of the two reported HIV-1 Group P viruses suggest that zoonosis occurred in the last 170 years and further support that pandemic HIV-1 Group M strains are better adapted to humans than non-pandemic or rare Group O, N and P viruses. The inability to antagonize human tetherin may potentially explain the limited spread of HIV-1 Group P in the human population.

## Background

The determinants of successful cross-species transmissions of primate lentiviruses are poorly understood, and may be critical in developing successful preventive strategies against primate lentiviral zoonoses in the future. Besides environmental factors, the adaptation of the virus to the immune system of its new host may determine its spread after zoonotic transmission. Mammalian restriction factors are interferon-inducible members of the innate immune system, and include the proteins TRIM5α and APOBEC3G/F [1-3]. These proteins evolved under positive selection pressure and provide barriers to cross-species transmission. The high variability of primate lentiviruses, however, may allow them to become resistant to TRIM5α through capsid sequence variation [4], and to gain activity against the restriction factors in the new host by the acquisition of mutations in their accessory genes. Tetherin, the most recently described restriction factor expressed by all major cellular targets of HIV infection (including CD4+ lymphocytes), is a transmembrane protein found at the plasma membrane, with an extracellular domain bearing a GPI anchor at its C-terminus [5]. This unusual topology with two membrane anchors enables tetherin to tether budding viruses to the infected cell, thus preventing their release, with subsequent internalization and degradation [6-9]. The delicate balance between antiviral restriction factors and viral escape is highlighted by the fact that single amino acid changes in viral or mammalian proteins may lead to gain or loss of function [10-13].

Most simian immunodeficiency viruses (SIVs), including SIVcpz and SIVgor that are found in chimpanzees and gorillas (reviewed in [14]), and represent the direct precursors of HIV-1, use Nef to antagonize tetherin [15-20], whilst SIV infecting certain monkeys (SIVgsn, SIVmus, SIVmon) use Vpu [17,21]. Human tetherin, however, is resistant to Nef due to a deletion in its cytoplasmic tail [15-20]. During its adaptation to humans, HIV-1 Group M has perfectly mastered this hurdle by switching from Nef to Vpu to antagonize human tetherin [16,17]. In contrast, Vpu proteins from non-pandemic Group O viruses do not antagonize tetherin and those of the rare Group N viruses gained some modest anti-tetherin activity but do not degrade CD4 [17,21]. The finding that pandemic HIV-1 M strains counteract human tetherin substantially more effectively than non-pandemic or rare Group O and N viruses suggests that effective tetherin antagonism may have been required for the efficient global spread of HIV/AIDS. The recent identification of a fourth group of HIV-1, designated HIV-1 Group P, that is closely related to SIVgor and most likely resulted from an independent gorilla-human transmission [22,23], provides another interesting opportunity to evaluate a possible correlation between effective tetherin antagonism and viral spread. Thus far, only two HIV-1 Group P strains have been described. The first one (RBF168) was isolated from a woman who had lived near Yaoundé, Cameroon [22]. The second (06CMU14788) was identified in a stored serum sample from a male patient admitted to Yaoundé hospital in 2006 [23]. In the present study, we performed phylogenetic analyses to assess how long Group P viruses may have been circulating in the human population and examined whether lack of anti-tetherin activity and thus suboptimal adaptation to humans may explain their limited spread in the human population.

## Results

### HIV-1 Group P entered the human population between 1845 and 1989

**Figure 1:**
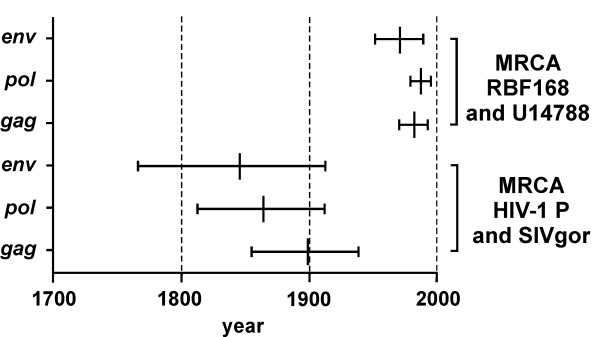
**Phylogenetic Analyses of HIV-1 Group P**. Bayesian Markov Chain Monte Carlo (MCMC) mean estimates of the year at which SIVgor and HIV-1 Group P (lower panel) as well as the two Group P sequences (upper panel) diverged from each other, based on the *gag, pol and env *loci. Horizontal bars represent the 95% Higher Density Probability (HDP). MRCA - Most Recent Common Ancestor. Data are representative of two independent simulations, all achieving convergence.

To determine whether HIV-1 Group P may have a relatively recent origin, we estimated the likely date of its zoonotic transmission by dating the HIV-1 Group P/SIVgor time of most recent common ancestor (tMRCA), as well as the MRCA of RBF168 and 06CMU14788. This analysis was performed using the Bayesian Markov chain Monte Carlo (MCMC) framework [24] implemented in the package BEASTv1.5.2. [25], using *gag, pol *and *env *gene sequences. We found good agreement in the HIV-1 Group P/SIVgor tMRCA estimates (Figure 1), obtained on the basis of the *gag *(1899 [95% Higher Probability Density: 1855-1938]), *pol *(1864 [1813-1912]) and *env *genes (1845 [1766-1912]). We also assessed the impact of including partial *gag *and *pol *sequences, of 1056 and 891 nucleotides respectively, from a more divergent SIVgor strain (BQ664) for which full-length sequence is not available [26]. No significant changes in the HIV-1 Group P/SIVgor tMRCA estimates were observed (data not shown). The tMRCA for RBF168 and 06CMU14788 was also estimated at each of the three loci (Figure 1), and gave consistent results as follows: *gag *1982 (1970-1992), *pol *1989 (1980-1995) and *env *1971 (1951-1988). Based on these analyses, we estimate that zoonosis occurred between 1845 and 1989. Sequences from additional HIV-1 Group P viruses and SIVgor strains would be required for more precise estimates. HIV-1 Group M is thought to have entered the human population around 1908 (1884-1924), Group O between 1905 and 1920 (1866-1940) [27,28], and Group N around 1963 (1948-1977) [28,29]. Thus, the four independent transmission events that gave rise to HIV-1 Groups M, O, N and P most likely all occurred relatively recently, i.e. within the last 170 years.

### HIV-1 P Group Vpu and Nef downregulate CD4 cell surface expression

**Figure 2:**
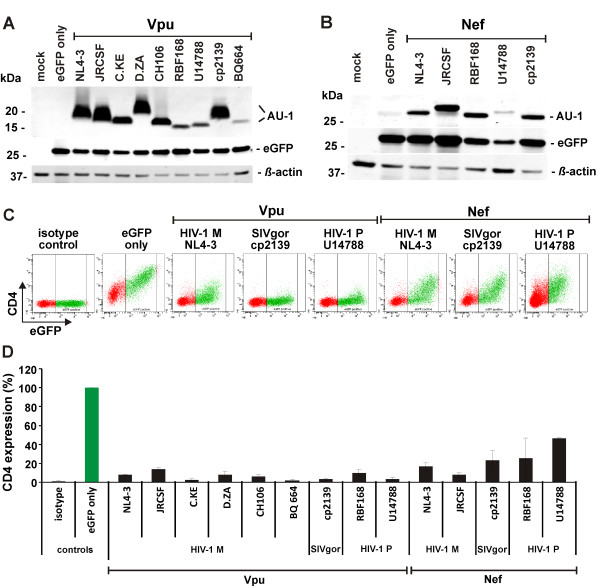
**Reduction of CD4 Surface Expression by HIV-1 Group P Vpu and Nef**. (A and B) Western blot analysis of cell lysates following transfection of 293T cells with pCGCG plasmids expressing eGFP alone (eGFP only) or together with the indicated AU-1-tagged Vpu or Nef proteins. Cell lysates were probed with anti-AU-1, anti-GFP and anti-beta-actin antibodies. (C) FACS analysis of 293T cells cotransfected with a CD4 expression vector and pCGCG plasmids expressing eGFP alone (GFP only) or together with the indicated *vpu *or *nef *alleles. (D) Reduction of Vpu- and Nef-mediated CD4 expression in 293T cells. Shown are the cell surface expression levels of CD4 relative to those measured in cells transfected with the eGFP only control vector. The range of eGFP expression used for the calculation is indicated in (C). The mean (± SD) of two independent experiments is shown.

We next assessed the ability of the two HIV-1 Group P viruses to antagonize gorilla and human tetherin as an indicator of adaptation to humans. To this end, the *vpu *or *nef *genes from HIV-1 P RBF168 and 06CMU14788 as well as two SIVgor strains (cp2139 and BQ644) were cloned into the bi-cistronic CMV-based pCGCG expression vector coexpressing the enhanced version of green fluorescent protein (eGFP) [30] and fused to a C-terminal AU1-tag. As further controls, we included *nef *and *vpu *alleles from five divergent HIV-1 M strains representing subtypes B (NL4-3, JR-CSF, founder virus CH106), C (C.KA) and D (D.ZA). Western blot analyses showed that all Vpu and Nef proteins were expressed at detectable levels (Figure 2A, B). Of note, our blots are consistent with previous observations that some Vpu proteins tend to aggregate and that the migration pattern on Western blots does not always reflect the calculated molecular weight [17]. Next, we determined whether the HIV-1 P Vpu and Nef proteins reduce cell surface expression of CD4. To measure this, 293T cells were cotransfected with vectors coexpressing *vpu *or *nef *and eGFP (or eGFP alone for control) together with a human CD4 expression construct, as previously described [17,31,32]. In the absence of Vpu or Nef, the cells expressed high levels of CD4 (Figure 2C). However, coexpression of the HIV-1 P Vpu decreased CD4 surface expression by more than 90% and HIV-1 P Nef reduced CD4 levels by 55 to 75% (Figure 2D).

### HIV-1 P Group Vpu and Nef are unable to counteract tetherin

**Figure 3:**
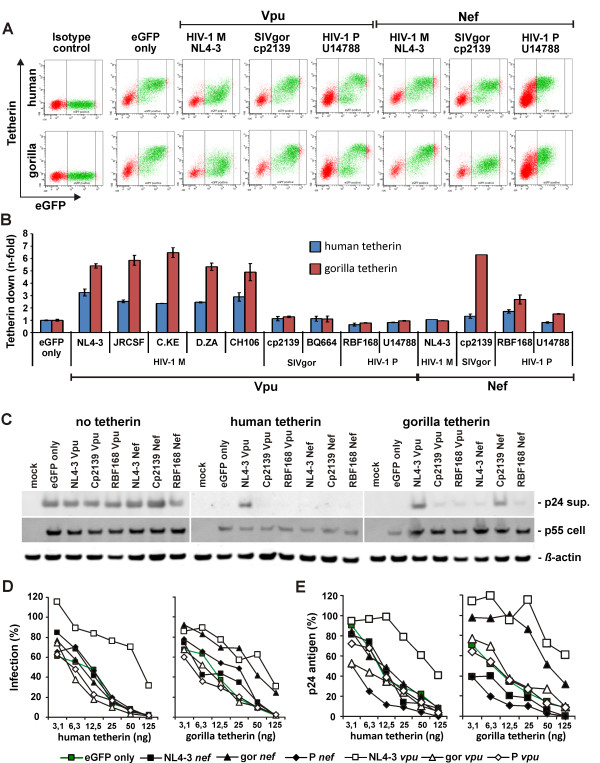
**Tetherin Counteraction by HIV-1 Group P Vpu and Nef**. (A) FACS analysis of 293T cells cotransfected with a human or gorilla tetherin expression vector and pCGCG plasmids expressing GFP alone (GFP only) or together with the indicated *vpu *or *nef *alleles. (B) Reduction of Vpu- and Nef-mediated tetherin expression in 293T cells. Shown is the n-fold reduction of tetherin cell surface expression levels relative to those measured in cells transfected with the eGFP only control vector. The range of GFP expression used for the calculation is indicated in panel A. The mean (± SD) of two independent experiments is shown. (C) Western blot analysis of cell and virion lysates following cotransfection of 293T cells with a *vpu*- and *nef*-defective proviral HIV-1 NL4-3 construct, pCGCG plasmids expressing eGFP alone (eGFP only) or together with the indicated *vpu *or *nef *alleles and an empty vector (no tetherin) or human or gorilla tetherin expression plasmids. Cell and virion lysates were probed with an anti-HIV-1 capsid p24 monoclonal antibody. Sup., cell culture supernatant. (D) Infectious virus and (E) p24 release from 293T cells following cotransfection with a *vpu*- and *nef*-defective proviral HIV-1 NL4-3 construct, pCGCG plasmids expressing eGFP alone (eGFP only) or together with the indicated *vpu *or *nef *alleles and different amounts of human or gorilla tetherin expression plasmids. Infectious virus was determined by infection of TZM-bl reporter cells and p24 release was quantified by ELISA. Values are shown as percentage of values obtained in the absence of tetherin. All infections were performed in triplicates. The mean of two independent experiments is shown.

We next tested the activity of Group P Vpu and Nef proteins against human and gorilla tetherin. To this end, we determined the levels of tetherin surface expression and infectious virus yields from HEK 293T cells cotransfected with a *vpu*/*nef*-deleted (Δ*vpu*Δ*nef*) HIV-1 Group M NL4-3 proviral construct [33], a tetherin expression plasmid, and a vector expressing Vpu or Nef [17]. As expected, HIV-1 Group M Vpus downregulated human tetherin about 5- to 6-fold (Figure 3A, B). In contrast, both HIV-1 Group P *vpu *alleles did not reduce tetherin cell surface expression (Figure 3B). This is not surprising since HIV-1 Group P Vpus lack the AxxxAxxxW transmembrane motif known to be critical for tetherin antagonism by HIV-1 Group M Vpus and may thus not interact with this restriction factor [34] (Additional file 1). Expression of HIV-1 P RBF168 and 06CMU14788 Nef did not affect surface expression of human tetherin either, but slightly decreased gorilla tetherin surface expression by 1.5- to 2.5-fold (Figure 3B). Unlike the SIVgor Nef, however, both HIV-1 Group P Nef proteins were unable to increase virion release in the presence of gorilla tetherin (Figure 3C-E and Additional file 2). This observation is consistent with reports that Vpu-mediated downmodulation of tetherin and viral release may be separable activities [35,36], although a similar finding has not yet been reported for Nef. Importantly, neither HIV-1 Group P Vpu nor Nef proteins were able to increase infectious virion or p24 release in the presence of human tetherin (Figure 3C-E and S2). This lack of anti-tetherin activity of Nef and Vpu shows that Group P viruses are not yet optimally adapted to the human host.

### Group P Envs reduce CD4 cell surface expression but do not counteract tetherin

**Figure 4:**
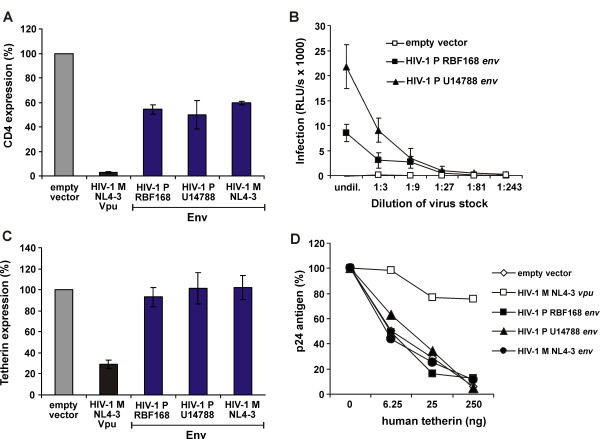
**Tetherin Counteraction by HIV-1 Group P Env**. (A) FACS analysis of 293T cells cotransfected with a CD4 expression vector and pCAGGS plasmids expressing the indicated *env *alleles or an empty vector control. Shown are the cell surface expression levels of CD4 relative to those measured in cells transfected with the vector control. The mean (± SD) of two independent experiments is shown. (B) Infectivity of virions pseudotyped with HIV-1 P Env. TZM-bl reporter cells were infected with different dilutions of the supernatant of 293T cells cotransfected with a *vpu*-, *nef*- and *env*-deficient HIV-1 NL4-3 proviral construct and the indicated *env *alleles. Infection was performed in triplicates and results were confirmed in three additional experiments. (C) FACS analysis of 293T cells cotransfected with a human tetherin expression vector and pCAGGS plasmids expressing the indicated *env *alleles or an empty vector control. Shown are the cell surface expression levels of tetherin relative to those measured in cells transfected with the vector control. The mean (± SD) of two independent experiments is shown. (D) p24 release from 293T cells following transfection with a *vpu*-, *env*- and *nef*-defective proviral HIV-1 NL4-3 construct, pCAGGS plasmids expressing the indicated *env *alleles and different amounts of human tetherin expression plasmids. p24 release was quantified by ELISA. Values are shown as percentage of values obtained in the absence of tetherin. The mean of two independent experiments is shown.

Besides Nef and Vpu, Env is the third primate lentiviral protein known to display anti-tetherin activity [11,37]. Thus, we also assessed the ability of HIV-1 Group P Env to antagonize tetherin. For this, both HIV-1 Group P *env *genes were chemically synthesized and cloned into the pCAGGS expression vector. Functional expression was assessed by measuring the levels of CD4 on 293T cells cotransfected with CD4 and Env expression constructs. We found that Group P Envs reduced CD4 surface expression levels about 2-fold, which is similar to the Group M NL4-3 Env (Figure 4A). We further demonstrated expression of functional RBF168 and 06CMU14788 *env *gene products by successful pseudotyping of an *env*-deficient HIV-1 M NL4-3 proviral construct and subsequent infection of TZM-bl reporter cells (Figure 4B).

We then tested antagonism of human tetherin by cotransfecting a *vpu*/*nef/env*-deleted (D*vpu*D*nef*D*env*) HIV-1 NL4-3 proviral construct along with RBF168 and 06CMU14788 Env in the presence of increasing amounts of tetherin expression plasmid. Both HIV-1 Group P Envs were unable to downregulate human tetherin from the cell surface (Figure 4C) and failed to increase virion release in the presence of human tetherin (Figure 4D). Since a previous study has shown that HIV-2 Env only antagonizes endogenous tetherin in Hela cells, but not transiently expressed tetherin in HEK 293T cells [37], we transfected Env expression plasmids along with the D*vpu*D*nef*D*env *HIV-1 NL4-3 proviral construct in HeLa cells and still found no effect of Group P Env on viral release (data not shown).

## Discussion

Previous studies suggest that effective tetherin antagonism may have been a prerequisite for the effective global spread of HIV/AIDS [16,17]. The human tetherin orthologue contains a deletion in the N-terminal cytoplasmic tail that renders human tetherin resistant to the accessory viral Nef protein. Nef is used by most primate lentiviruses, including SIVcpz, SIVgor and SIVsmm, the direct precursors of HIV-1 and HIV-2, to counteract tetherin in their respective host species. Thus, tetherin represents a significant barrier to effective zoonotic transmission of primate lentiviruses [15-20]. Pandemic HIV-1 Group M strains fully cleared this hurdle by switching from Nef to Vpu to counteract tetherin in the new human host [17]. In contrast, the non-pandemic Group O viruses seem to be unable to antagonize human tetherin and the rare Group N viruses gained only modest anti-tetherin activity and lost the second major function of Vpu, i.e. degradation of CD4, during adaptation to humans [17,21]. The recent description of a fourth group of HIV-1, designated "P", that most likely represents an independent cross-species transmission of SIVgor infecting gorillas to humans [22,23], allowed us to further investigate a possible role of effective tetherin antagonism in the spread of the different groups of HIV-1. In the present study, we thus determined whether or not HIV-1 Group P has cleared the tetherin barrier in humans.

To assess whether the transmission event that resulted in HIV-1 Group P also occurred relatively recently, as previously described for Group M, O and N viruses [27-29], we first performed extensive phylogenetic analyses. Our results suggest that Group P entered the human population between 1845 and 1989. This window is rather wide due to the fact that only two HIV-1 Group P strains have been described to date [22,23] and could be further narrowed once additional Group P or SIVgor sequences become available. It is also noteworthy that the possibility that the Group P zoonosis did not result from a gorilla, but from a yet-to-be-identified chimpanzee population can currently not be entirely dismissed [22].

In spite of the aforementioned limitations, our analyses suggest that the transmission of SIVgor that gave rise to HIV-1 Group P occurred within the last 170 years and is thus similar to three zoonotic transmission events that led to the emergence of HIV-1 Groups M, O and N [27-29]. Our functional analyses demonstrate that the *vpu *and *nef *genes of both HIV-1 Group P strains are unable to antagonize human tetherin. These results are in agreement with a recent study reporting lack of anti-tetherin activity of Group P Vpu and Nef proteins [38], published while this manuscript was under revision. Our analyses significantly expand these findings because only the RBF168 strain was examined in the previous study, whereas we also examined the Vpu and Nef proteins of the second HIV-1 Group P 06CMU14788 strain. Perhaps even more importantly, we also show that the Env proteins of both HIV-1 Group P strains do not antagonize tetherin, although they are otherwise active and capable of mediating HIV-1 entry. These results strongly suggest that HIV-1 Group P has not yet evolved an effective antagonist of human tetherin. A recent origin and suboptimal adaptation to the new human host is further supported by the fact that only one of the two HIV-1 Group P strains (06CMU14788) has evolved a human-specific change of M30K in the viral p17 matrix protein that is present in all other groups of HIV-1 and seems to be relevant for the replicative fitness of HIV-1 in the human host [39].

We found that the HIV-1 Group P Nef was unable to promote virion release although it reduced the levels of cell surface expression of the gorilla tetherin orthologue by up to 2-fold. This result is in agreement with recent data on Vpu function showing that promotion of virus release and downmodulation of tetherin from the cell surface do not always correlate [35,36]. A possible reason for this is that effective tetherin antagonism by Vpu and/or Nef may involve several distinct but cooperative mechanisms [16,40]. For Vpu, it has been reported that it interacts directly with the transmembrane domain of tetherin, sequesters the restriction factor away from the sites of viral budding to the trans-Golgi network and the perinuclear compartment and induces its proteasomal and/or lysosomal degradation [41-43]. The mechanism of Nef-mediated tetherin antagonism is poorly investigated and may involve AP-2 dependent removal of tetherin from the sites of virion assembly [44]. It is tempting to speculate that some Vpu and Nef proteins may be able to down-modulate tetherin from the cell surface and/or to increase its degradation but lack the capability to sequester tetherin away from the sites of virion budding and are thus unable to efficiently promote the release of progeny virions.

## Conclusions

In conclusion, our data further support that effective tetherin antagonism may play a role in the effective spread of HIV/AIDS. However, further studies using replication-competent full-length molecular clones of HIV-1 Group O, N and P strains are required to exclude the possibility that they may counteract the tetherin restriction through an as yet unknown mechanism. It is evident that HIV-1 Groups O and N can cause AIDS [45,46], whereas the pathogenic potential of Group P remains to be determined. Furthermore, it remains elusive whether the viral loads, rates of CD4 T cell decline and clinical progression, or the efficiency of virion shedding into genital fluids, differ between individuals infected with pandemic HIV-1 Group M and non-pandemic Group O or rare Group N and P strains. In-depth studies of the virological, immunological and clinical characteristics of Group O, N and P infections in humans may provide important insights into virus transmission and seem highly warranted.

## Methods

### Dating the most recent common ancestor of HIV-1 Group P and SIVgor

Full-length genome sequences representative of HIV-1 and related SIV strains were retrieved from Genbank, together with the corresponding sampling dates. The dataset included four SIVcpz sequences from *Pan troglodytes schweinfurthii *(GenBank accession numbers: :AF447763; EF394357; EF394358; U42720), ten SIVcpz sequences from *Pan troglodytes troglodytes *(AF103818; AF115393; AJ271369; AY169968; DQ373063; DQ373064; DQ373065; DQ373066; EF535994; X52154), four SIVgor sequences from *Gorilla gorilla gorilla *(FJ424863; FJ424864; FJ424865; FJ424871); eight reference HIV-1 Group M sequences from subtype A (AB253429), subtype B (K03455), subtype C (U46016), subtype D (K03454), subtype F (AJ249238), subtype H (AF190127), subtype J (AF082394) and subtype K (AJ249235); four HIV-1 Group N sequences (AJ006022; AJ271370; AY532635; DQ017383); four HIV-1 Group O sequences (AJ302647; AY169812; L20571; L20587); two HIV-1 Group P sequences available to date (GQ328744; HQ179987). A multiple nucleotide sequence alignment was created using the program MUSCLE version 3.6 http://www.drive5.com/muscle/download3.6.html, and manually edited with the program Se-Al http://tree.bio.ed.ac.uk/software/seal/. Regions of the alignment that could not be unambiguously aligned where excluded from the analysis. Dated phylogenies and time estimates were initially obtained for the *gag, pol *and *env *loci under various evolutionary and demographic assumptions.

The full-length, *gag, pol*, and *env *sequence alignments were used to reconstruct the phylogenies of the selected viruses using a Bayesian Markov chain Monte Carlo (MCMC) framework. A posterior distribution of phylogenetic trees was obtained for each alignment using the software MrBayes version 3.1.2 http://mrbayes.csit.fsu.edu/download.php, under the General Time Reversible model of nucleotide substitution with proportion of invariable sites and gamma-distributed rate heterogeneity (GTR+I+Γ). The MCMC search was set to 10,000,000 iterations, with trees sampled every 1000^th ^generation. Convergence of the estimates was checked using the program Tracer (i.e. effective sampling size > 200). A model incorporating the SRD model for nucleotide substitution, where the evolution of 3^rd ^codon positions is modeled independently to 1^st ^and 2nd codon positions, a relaxed molecular clock model [47] and a Bayesian Skyline coalescent prior [48] fitted the data significantly better than alternative models (Bayes factor > 20).

### Cloning and viral constructs

RBF168 and 06CMU14788 Vpu (accession nos: GQ328744 and HQ179987) and Nef (accession nos: GQ328744 and HQ179987) were synthesized by Genscript, C-terminally tagged with an AU1-tag and cloned into the pCGCG vector coexpressing eGFP via an IRES [17]. RBF168 and 06CMU14788 Envelopes were also synthesized by Genscript and cloned into the pCAGGS vector as previously described [11]. Human and gorilla tetherin were cloned into the pCGCG vector coexpressing DsRed2. All experiments were performed in transfected 293T that overexpress tetherin, GFP and Nef, Vpu or Env after transfection with the CMV- or chicken β-actin promoter based pCGCG or pCAGGS expression constructs. These plasmids have been previously described [17,32,49].

### CD4 cell surface staining and flow cytometry

293T cells were cotransfected with human CD4 (0.5 μg) and different *vpu, nef *or *env *alleles (2.5 μg) or GFP only. 48 h hours post transfection CD4 surface expression was measured by flow cytometry as previously described [17,32].

### Viral release assay, p24 ELISA, and Western blotting

293T cells were cotransfected with pBR-NL43 Δ*nef*Δ*vpu *(2.0 μg), different amounts of tetherin (0-125 ng) and different *vpu *or *nef *alleles (0.5 μg). 48 hours post infection virus release was determined by infection of TZM-bl reporter cells. Values were normalized (no tetherin = 100%) [17]. p24 ELISA and Western blot were carried out as previously described [17,32]. For the verification of activity of P Group Env a pBR-NL43 Δ*nef*Δ*vpu*Δ*env *expression vector was used. TZM-bl reporter cells were used to analyze infectivity of HIV-1 Group P Env pseudotyped virions (NIH AIDS Research and Reference Reagent Program).

## Competing interests

The authors declare that they have no competing interests.

## Authors' contributions

DS participated in the design of the study, performed most of the experiments and contributed to the final manuscript. SH coordinated the phylogenetic studies. SJP performed some of the experiments. JCP and GJT helped to conceive the study and participated in the review of the manuscript. RKG conceived and coordinated the study, carried out the phylogenetic analyses and wrote the draft manuscript. FK participated in the design and coordination of the study and wrote the final manuscript. All authors read and approved the final manuscript

## Supplementary Material

Additional file 1 **Alignment of SIVgor, HIV-1 M and P Vpus**. Amino acid alignment of Vpu proteins from HIV-1 Group M, Group P and SIVgor. An AxxxAxxxW transmembrane motif that has been shown to be crucial for tetherin antagonism by HIV-1 Group M Vpus is highlighted in yellow. Click here for file

Additional file 2 **Tetherin Counteraction by HIV-1 Group P Vpu and Nef**. (A) Infectious virus and (B) p24 release from 293T cells following cotransfection with a *vpu*- and *nef*-defective proviral HIV-1 NL4-3 construct, pCGCG plasmids expressing eGFP alone (eGFP only) or together with the indicated *vpu *or *nef *alleles and a human or gorilla tetherin expression plasmid. Infectious virus was determined by infection of TZM-bl reporter cells and p24 release was quantified by ELISA. Values are shown as percentage of values obtained in the absence of tetherin. All infections were performed in triplicates. The mean of two independent experiments is shown. Click here for file
