# Supplementary figures and images for: HIV-1 Group P is unable to antagonize human tetherin by Vpu, Env or Nef
Source: Retrovirology. 2011 Dec 15;8:103. doi: 10.1186/1742-4690-8-103 (PMC3285029; doi:10.1186/1742-4690-8-103)

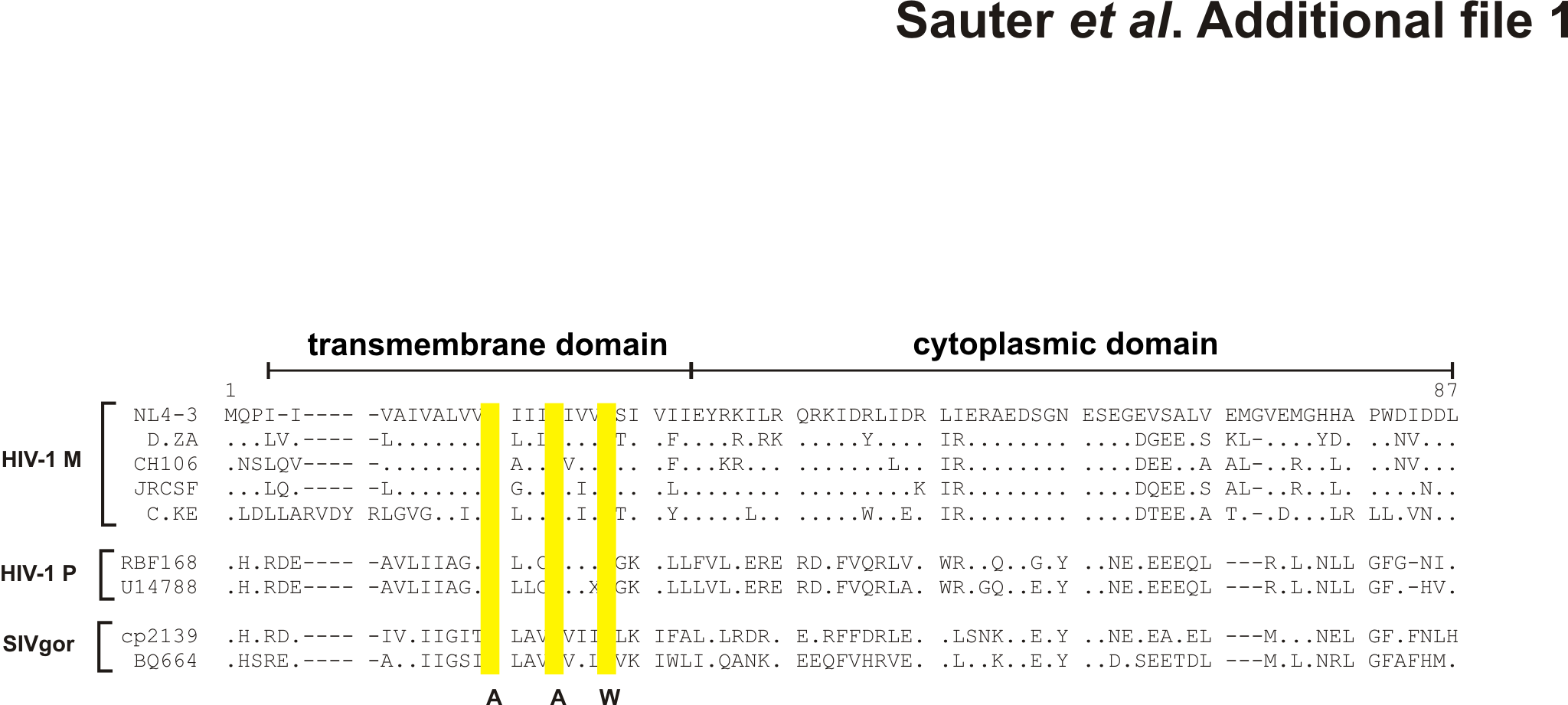

Supplement: Additional file 1 — Alignment of SIVgor, HIV-1 M and P Vpus. Amino acid alignment of Vpu proteins from HIV-1 Group M, Group P and SIVgor. An AxxxAxxxW transmembrane motif that has been shown to be crucial for tetherin antagonism by HIV-1 Group M Vpus is highlighted in yellow. [file 1742-4690-8-103-S1.TIFF]

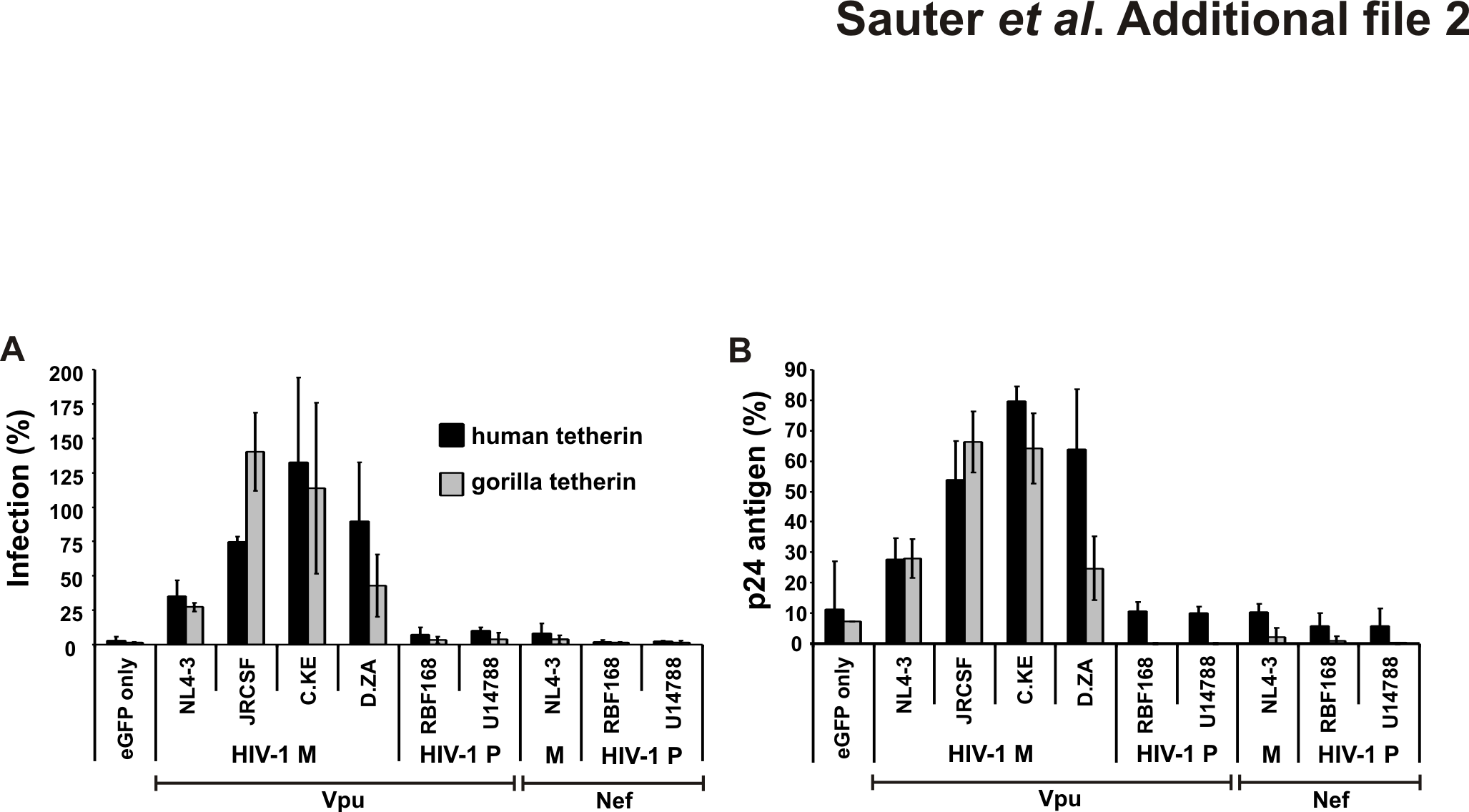

Supplement: Additional file 2 — Tetherin Counteraction by HIV-1 Group P Vpu and Nef. (A) Infectious virus and (B) p24 release from 293T cells following cotransfection with a vpu- and nef-defective proviral HIV-1 NL4-3 construct, pCGCG plasmids expressing eGFP alone (eGFP only) or together with the indicated vpu or nef alleles and a human or gorilla tetherin expression plasmid. Infectious virus was determined by infection of TZM-bl reporter cells and p24 release was quantified by ELISA. Values are shown as percentage of values obtained in the absence of tetherin. All infections were performed in triplicates. The mean of two independent experiments is shown. [file 1742-4690-8-103-S2.TIFF]
